# A hybrid deep learning framework for bacterial named entity recognition with domain features

**DOI:** 10.1186/s12859-019-3071-3

**Published:** 2019-12-02

**Authors:** Xusheng Li, Chengcheng Fu, Ran Zhong, Duo Zhong, Tingting He, Xingpeng Jiang

**Affiliations:** 1 School of Computer, Central China Normal University, Wuhan, Hubei China; 2 Hubei Provincial Key Laboratory of Artificial Intelligence and Smart Learning, Central China Normal University, Wuhan, Hubei China; 3 Collaborative & Innovation Center, Central China Normal University, Wuhan, Hubei China

**Keywords:** Named entity recognition, Biomedical text mining, Conditional random field, Deep learning

## Abstract

**Background:**

Microbes have been shown to play a crucial role in various ecosystems. Many human diseases have been proved to be associated with bacteria, so it is essential to extract the interaction between bacteria for medical research and application. At the same time, many bacterial interactions with certain experimental evidences have been reported in biomedical literature. Integrating this knowledge into a database or knowledge graph could accelerate the progress of biomedical research. A crucial and necessary step in interaction extraction (IE) is named entity recognition (NER). However, due to the specificity of bacterial naming, there are still challenges in bacterial named entity recognition.

**Results:**

In this paper, we propose a novel method for bacterial named entity recognition, which integrates domain features into a deep learning framework combining bidirectional long short-term memory network and convolutional neural network. When domain features are not added, F1-measure of the model achieves 89.14%. After part-of-speech (POS) features and dictionary features are added, F1-measure of the model achieves 89.7%. Hence, our model achieves an advanced performance in bacterial NER with the domain features.

**Conclusions:**

We propose an efficient method for bacterial named entity recognition which combines domain features and deep learning models. Compared with the previous methods, the effect of our model has been improved. At the same time, the process of complex manual extraction and feature design are significantly reduced.

## Background

Microorganisms are ubiquitous in nature. Human beings are exposed to microorganisms from birth to death and are associated with microorganisms during all stages of life. The human body together with its microbiome constitutes a super-species, forming our own exclusive microbial community [1]. Studies have shown that microbial diversity is associated with various human diseases, including allergy, diabetes, obesity, arthritis, inflammatory bowel disease, and even neuropsychiatric diseases [2–4]. Therefore, the diversity of microbial communities and the interaction between microorganisms and the host immune system play crucial role in guaranteeing human healthy. Microorganisms in microbial communities interact with other members actively which ensures the stability and diversity of microbial communities [5]. Thus it is important to explore the microbial interaction for understanding the structure of microbial community and applying these results to the biomedical field. In the past, the method of extracting microbial relationships traditionally is to culture bacteria separately in biological laboratory. However, most microbes cannot be cultured experimentally as well as it is time-consuming and expensive. Recently, computational approaches can alleviate above problems to some extent thanks to the development of high-throughput sequencing technologies. At present, there are several kinds of computational methods for this task including exploring microbial interactions from metagenomic data, inferring microbial interaction from genomic information and mining microbial interaction from biomedical literature [5]. The two former computational approaches are widely explored; however, extracting the microbial interaction from the biomedical literature is less popular. There are rich relevant researches published in the literature confirming certain microbial interactions through direct experiments. It will be a valuable resource to explore the microbial interaction by mining biomedical literatures and integrate these knowledge into a database or knowledge graph. Nevertheless, the rapid growth in the volume of biomedical literature and the variety of microorganisms make manual interaction extraction barely possible.

In previous work, Freilich [6] proposed a microbial interaction extraction method based on the co-occurrence model. They first extracted the species names from the intestinal microbial abundance data. Then, they retrieved articles with the two species in PubMed and calculated the co-occurrence probability of the species. Finally, a microbial co-occurrence network was constructed to predict microbial interaction. Similarly, Lim [7] obtained the data in the same way and put forward an automated microbial interaction extraction method based on support vector machine (SVM). What they had in common was the process to get the species from microbial abundance data of the human gut, which might result in the omission of certain potential interactions due to the different standards of spelling species names.

In recent years, with the development of natural language processing (NLP), text mining strategy makes it possible to extract microbial interaction from unstructured texts. Furthermore, named entity recognition (NER) is the core task of interaction extraction (IE). The purpose of NER is to extract words with special meaning from the text, such as *Person*, *Location*. Various methods about NER have been proposed as the advancement of computer technology, which are mainly based on following three categories:(1) rule-based method [8]; (2) machine learning-based method [9], 3) neural network-based method [10]. It is not portable and universal that rule-based way needs to design rules in specific domain with experts. The second approach based on statistical machine learning has strong portability and excellent performance, but it requires complex feature engineering and large-scale labeling. Furthermore, neural network based method has the highlighting performance without cumbersome process of feature design as well as large-scale tagging data. Although the method of NER in the general domain has fully developed, it is a challenging task in the domain of bacterial name identification on account of complexity of microbial names.

Wang [11, 12] proposed a method of bacterial named entity recognition based on conditional random fields (CRF) and dictionary, which contains more than 40 features (word features, prefixes, suffixes, POS, etc.). The model effect was optimized after selecting the best combinations of 35 features, in the meanwhile, the computing efficiency of this model was greatly improved by deploying the model on Spark platform. Unfortunately, CRF and dictionary-based method need manually design features and additionally dictionary resources, and the result of the model depend on the quality of the annotated data and the rationality of the feature design.

In the last few years, deep learning has been widely utilized and has achieved great performance in many fields, such as image [13]; speech recognition [14]; machine translation [15]; reading comprehension [16] and so on. Similarly, the method based on deep learning has attracted extensive attention in the field of NER. Lample [17] first adopted Bi-LSTM -CRF for NER, Ma [18] introduced Bi-LSTM-CNN- CRF for NER, in which CNN was used to extract character-level features. Since then, more and more deep learning algorithms are used for NER. Also, the biomedical text mining contest was organized to accelerate the research on biomedical [19, 20], and many of top participating systems utilized deep learning in biomedical text [10, 21]. Li [22] shown that deep learning-based method could acquire well performance in bacterial NER. However, his work did not take advantage of the existing biological resources and incorporate them as features into the model.

In this paper, we propose a method combining domain features and deep learning for bacterial NER, which achieves excellent performance in dataset. When adopting POS features only, the F1-measure of the model reaches 89.4%. With POS features and dictionary features are both added, the F1-measure is up to 89.7%. The experimental results demonstrate that external resources can contribute to the improvement of the result of the model.

## Materials and methods

**Fig. 1:**
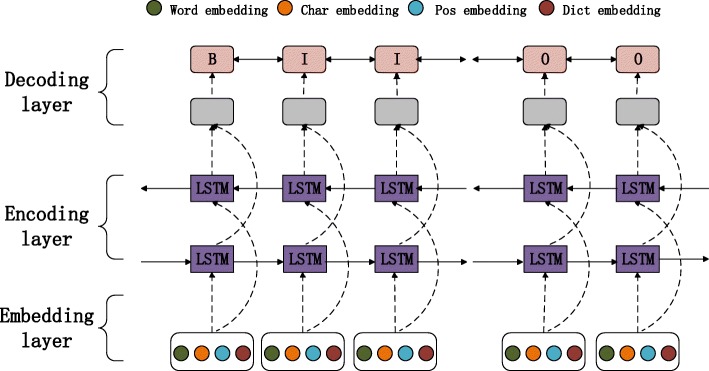
The model proposed in this paper. The concatenated word-level embedding, char-level embedding, pos embedding and dict embedding are input into encoding layer for learning, then the output of encoding layer are input into decoding layer for predict

As shown in Fig.1, we build a model mainly divided into the following three layers: embedding layer, encoding layer and decoding layer. Firstly, we concatenate pre-trained word embedding, character-level embedding extracted by convolution neural network, POS embedding and dictionary embedding and input it into the encoding layer. Then the encoding layer is used for parameter learning. In the end, we can predict the best output path of sentence through the decoding layer.

### Embedding layer

#### Word embedding

According to a recent study, word embedding has achieved outstanding results in the field of NLP. Compared with the traditional encoding method, the word embedding technique can fully exploit semantic information between words, for example “king” – “man” + “woman” = “queen”, as well as using a low-dimensional continuous vector to represent the vector of words. This not only solves the sparse problem of the vector, but also obtains semantic information of the word. Currently, there are some well-performed word embedding tools which are widely used, such as fastText [23], glove [24], Word2vec [25]. At the same time, Moen [26] pre-trained a word embedding PubMed2vec with word2vec in the field of biomedical text mining. In our work, in order to obtain higher quality of word vectors, we downloaded more than 400 thousand abstracts about bacteria from PubMed and then used them together with our corpus to train word vectors. We adopted the skip-gram model of word2vec provided in gensim [27] to train our corpus.

#### Char embedding

**Fig. 2:**
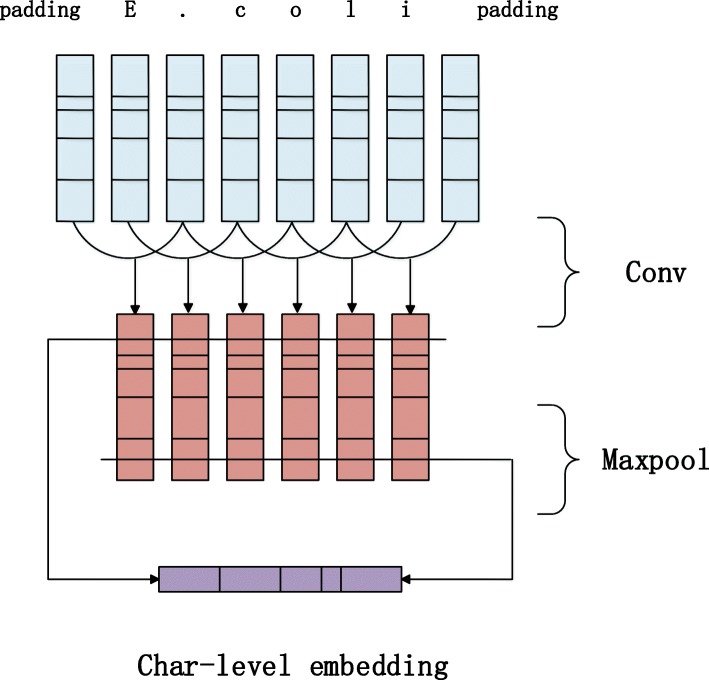
The method to get char-level embedding in our paper. The characters in a word are transfer to vectors, then though a convolution layer and a max-pooling, finally the output are concatenated to represent the word

As shown by previous studies, character-level features have been proved to be work well in many NLP tasks. Kim [28] used CNN to obtain character representation and then utilized LSTM to train a language model. Santos and Chiu [29] showed that CNN could extract word morphological features (prefix and suffix etc.) effectively and encoded them into neural network. Lample [17] also demonstrated that LSTM could extract morphological features of words. But, experiment results show that CNN is better than LSTM in the task of NER. As a consequence, in this paper, we use the CNN to obtain the character-level features of words. Figure 2 illustrates detailed process of our method. Given a word W= ${\left[{c}_i\right]}_0^T$, T is the length of sequence, *c*_*i*_ represents the character of the word, e(*c*_*i*_) is the character vector for each character. In order to acquire morphological features of words, we use N times of convolution kernels X to perform convolution operations. The size of convolution kernels is k. The calculation formula of *O*_*i*_ output for each convolution can be written as:

1 $$ {O}_i=\mathrm{relu}\left({W}_1{X}_i+{b}_1\right) $$

Where *W*_1_ denote the weight matrix and *b*_1_ denote the bias vector, *X*_*i*_ = [*e*(*c*_*i* − *k*_), …, *e*(*c*_*i*_), …, *e*(*c*_*i* + *k*_)], relu denote the activation function. Finally, for each convolution kernel output *O*_1_, …, *O*_*i*_, …, *O*_*N*_, the max-pooling operation is performed to obtain the character vector representation of the word. The j-th vector representing *W*_*j*_ can be computed as:

2 $$ {W}_j=\underset{1\le i\le N}{\max }{O}_{i_j} $$

#### Domain features

Inspired by the related work of Chiu [29] and Huang [30], some artificial designed features and domain knowledge can also promote the effectiveness of the neural network model. Consequently, in this paper, we discuss the influence of POS and dictionary features on the neural network model.

In fact, although the model of neural network can extract feature automatically to some extent, some linguistic features cannot be well learned on account of the complicacy of natural language processing. We use the nltk [31] tool to get the POS features of each word, and bidirectional maximum matching algorithm (BDMM) [32] to obtain dictionary features. UMLS [33] is a unified medical database, which contains volume of standardized names and abbreviations for diseases, proteins, genes and microorganisms. Hence we extract all the bacterial names from UMLS and integrate them into a bacterial dictionary. Table 1 gives an example of our preprocessing data.

**Table 1:** The example of the data format in our paper

sentence	pos	dict	tag
Actinobacillus	NNP	B-bacteria	B-bacteria
actinomycetemcomitans	NNS	I-bacteria	I-bacteria
,	,	O	O
Porphyromonas	NNP	B-bacteria	B-bacteria
gingivalis	NN	I-bacteria	I-bacteria
,	,	O	O
and	CC	O	O
Peptostreptococcus	NNP	B-bacteria	B-bacteria
micros	NNS	I-bacteria	I-bacteria

#### Encoding layer

The long short-term memory network is a [34] variant of recurrent neural network (RNN). It solves the problems of the gradient disappearance and the gradient explosion in the training process of RNN [35, 36]. In the practical application process, LSTM can handle the time series problem and the long-distance dependence problem well. It mainly consists of three gates: input gate, output gate and forget gate. The main formula is as follows:

3 $$ {f}_t=\sigma \left({W}_f\bullet \left[{h}_{t-1},{x}_t\right]+{b}_f\right) $$

4 $$ {i}_t=\sigma \left({W}_i\bullet \left[{h}_{t-1},{x}_t\right]+{b}_i\right) $$

5 $$ {o}_t=\sigma \left({W}_o\bullet \left[{h}_{t-1},{x}_t\right]+{b}_o\right) $$

6 $$ {C}_t={f}_t\ast {C}_{t-1}+{i}_t\ast \tanh \left(\mathrm{W}\bullet \left[{h}_{t-1},{x}_t\right]+b\right) $$

7 $$ {h}_t={o}_t\ast \tanh \left({C}_t\right) $$

Where *σ* denote sigmoid function, *x*_*t*_ denote the input of LSTM, *h*_*t*_ denote the output of LSTM, *W*_*f*_, *W*, *W*_*o*_, *W*_*i*_ denote the weight matrix in the process of training , *b*_*f*_, *b*_*i*_, *b*_*o*_, b is the bias vector.

For many sequence labeling tasks, we should consider the context information of the word at the same time, but a single LSTM structure can only obtain the historical information of the word. For this reason, Dyer [37] proposed a bidirectional long short-term memory (Bi-LSTM) network for acquiring the history information and future information of words. At first, given a sequence X= ${\left[{x}_t\right]}_0^n$, n represents the length of sequence, *x*_*t*_ is the input vector at time t, use a forward LSTM to obtain historical information $\overrightarrow{h_t}$ =LSTM (${\overrightarrow{h}}_{t-1}$, *x*_*t*_). Then a backward LSTM to obtain future information $\overleftarrow{h_t}= LSTM\left({\overleftarrow{h}}_{t+1},{x}_t\right)$. Finally, the outputs from both directions are concatenated to represent the word information ${h}_t=\left[\overrightarrow{h_t};\overleftarrow{h_t}\right]$ learned at time t.

#### Decoding layer

For the task of sequence labeling, we should consider the dependency problem between words, because the neighboring words of the current word contribute to the labeling of the word, so we introduce the conditional random fields (CRF) [38] on the top of encoding layer. CRF has been proved to have a good effect on sequence labeling. Given the input of a sentence:

8 $$ \mathrm{X}=\left({x}_1,\dots, {x}_i,\dots, {x}_n\right) $$

Where *x*_*i*_ denote the vector representation of the output of encoding layer. We define P as the score matrix output by Bi- LSTM, the size of the matrix P is n × m, n represents the length of the sentence, m is the number of types of output tags and *P*_*ij*_ represents the probability of the j-th tag of the i-th word. The output of the definition sentence is:

9 $$ \mathrm{y}\kern0.5em =\left({y}_1,\dots, {y}_i,\dots, {y}_n\right) $$

Where *y*_*i*_ represents the output prediction for each word. The score we define for the sentence is:

10 $$ \mathrm{S}\left(\mathrm{X},\mathrm{y}\right)=\sum \limits_{i=0}^n{T}_{y_{i,}{y}_{i+1}}+\sum \limits_{i=1}^n{P}_{i,{y}_i} $$

Where T represents the tag transition matrix, for example, *T*_*ij*_ represents the transition probability from tag i to tag j. *y*_0_ and *y*_*n* + 1_ denote the start and end that we add to the matrix, so the size of T is m + 2. T is learned during training. Then, softmax function is used to normalize the output path y:

11 $$ \mathrm{P}\left(\mathrm{y}|\mathrm{X}\right)=\frac{e^{s\left(X,y\right)}}{\sum \limits_{\overset{\sim }{y\in }Y}{e}^{S\left(X,,\overset{\sim }{y}\right)}} $$

Where Y is the set of all possible output sequences of sentence X, and we maximize log-probability of the correct output sequence during the training, which can represented as follows:

12 $$ \log \left(\mathrm{P}\left(\mathrm{y}|\mathrm{X}\right)\right)=\mathrm{S}\left(\mathrm{X},\mathrm{y}\right)-\log \left(\sum \limits_{\overset{\sim }{y}\in Y}{e}^{S\left(X,,\overset{\sim }{y}\right)}\right) $$

In the decoding stage, we predict the best output path through maximizing the score function:

13 $$ {\displaystyle \begin{array}{c}\mathrm{y}\ast = argmaxS\left(X,\overset{\sim }{y}\right)\\ {}\overset{\sim }{y}\in Y\end{array}} $$

This process can be implemented by dynamic programming and inferred by Viterbi algorithm [39].

#### Dataset

In this paper, we utilize the dataset proposed by Wang [11] . They used “bacteria”, “oral” and “human” as keywords to retrieve relevant abstracts from PubMed for nearly 10 years. At last they selected 1030 abstracts as train set and 314 abstracts as test set. The statistics about dataset are shown in Table 2. In order to evaluate the performance of the model, we divided it into training set, validation set and test set, in which 20% of the original training set was taken as validation set. We downloaded all abstracts related to “bacteria” from PubMed in the past decade and then trained word vectors along with the dataset.

**Table 2:** The statistics of the dataset in our experiment

Data set	abstract	sentence	token	The kind of entities	Entities	Entities token
Train set	1030	10094	252109	1767	7637	15272
Test set	314	3159	77638	770	2260	4611

#### Tagging scheme

In this experiment, our task is to give each word in the sentence a tag. As we investigated, a bacterial entity in a sentence may be composed of multiple words, so we need a set of identifiers to represent it. Currently, there are three main types of tagging scheme: IOB2, BIOE and BIOES. To compare the performance with other models, we use the IOB2 format as our tagging scheme. In the IOB2 tagging method, B-label represents the starting word of an entity, I-label represents the inside word of an entity, and O represents the word is not in entity.

#### Training and hyper-parameter settings

In this experiment, the following four parts constitute the input of our model: word embedding, character embedding, pos embedding, dict embedding. The word embedding is trained by word2vec with the dimension is 300, and the character embedding is trained by CNN. The initial input of the characters vector are 25-dimensional. The dimensions of the pos embedding and the dict embedding are 25, 5, respectively. The input embeddings all randomly initialized with uniform samples from $\left[\sqrt{-3/\mathit{\dim}},\sqrt{3/\mathit{\dim}}\right]$ where *dim* is the dimension of embeddings [40]. The convolutional layers and fully connect layers were initialized with glorot uniform initialization [41], bias vectors are initialized with 0. Then the four embeddings are concatenated to input the model for parameter learning. During the training, we use the back propagation algorithm to update the parameters. Our optimization function is Adam [42] algorithms with a learning rate of 0.001 and a decay rate of 0.9.

We introduce dropout [43] and early stopping [44] technology to the model during the process of training. The purpose of the dropout technique is to prevent over-fitting of the model by randomly dropping some hidden nodes during the training process. We introduce dropout technology both before and after the decoding layer, which set dropout rate = 0.5. The principle of early stopping technology is to stop training when the result of the validation set is no longer improved within a tolerance range class, and record the parameters of model which has best result. It can prevent over-fitting of the model and select the best iteration number effectively. In this experiment, we set patience = 5. The detailed parameters are shown in Table 3.

**Table 3:** The hyper-parameter in our experiment

Hyper-parameter	
word embedding	300
char embedding	25
pos embedding	25
dict embedding	5
filter size	3
filter deep	30
lstm hidden	100
Dropout	0.5

### Evaluation metrics

In order to evaluate the performance of the model proposed in this paper, we choose P (precision), R (recall) and F1 (F1-measure) as experiment metrics.

14 $$ \mathrm{P}=\frac{TP}{TP+ FP} $$

15 $$ \mathrm{R}=\frac{TP}{TP+ FN} $$

16 $$ \mathrm{F}1=\frac{2\times P\times R}{P+R} $$

Where TP is the number of entities correctly identified and FP is the number of non-entities identified as entities. F1-measure is the harmonic average of P and R.

## Results and discussion

The experimental results are shown in Table 4. Model 1 and Model 2 were proposed by Wang [11, 12], and their models were based on traditional machine learning methods. Therefore, they manually extracted 43 groups of features, and then achieved good results on the dataset through feature combination and selection. Besides, the model based on Spark was greatly improved in speed. The model we proposed previously was based on neural network and did not need to extract features manually [22]. It was an end-to-end model and had enhanced the effect of the bacteria NER to some extent, but it did not make full use of the linguistic features and existing resources. In this paper, we consider the influence of domain features on the model. The experimental results show that the F1-measure of the model achieves 89.4% when adding the POS feature. With dictionary features and POS features are added, the model’s F1-measure is up to 89.7%. From the above, we can include that these two features can effectively improve the effect of the model.

**Table 4:** The result of our model

Model	P	R	F
CRF and dictionary [11]	88.476%	81.149%	84.654%
spark [12]	89.443%	82.899%	86.047%
HDL_CRF [22]	90.009%	88.300%	89.146%
+pos	90.502%	88.344%	89.410%
+pos + dict	90.404%	89.007%	89.700%

**Fig. 3:**
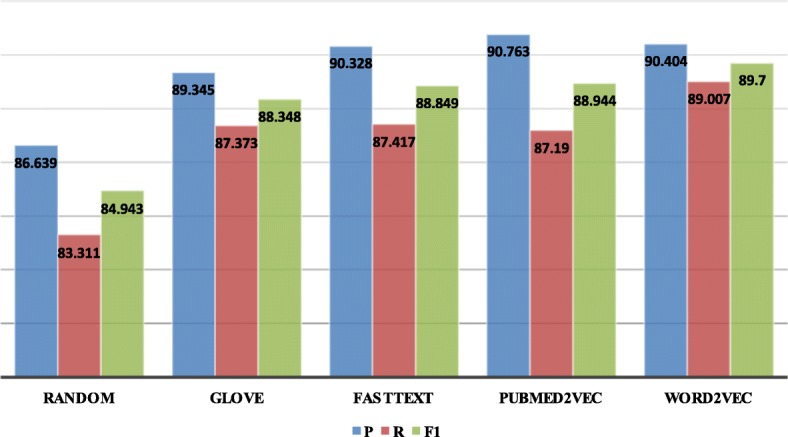
The influence of different embedding in model

In order to evaluate the impact of word embedding on the model, we compare the performance of four pre-trained word embedding: glove [24], fastText [23], word2vec [25] and PubMed2vec [26] as well as random initialization in our model. Among them, glove and fastText are trained on Wikipedia which the dimension are 300, Pubmed2vec is 200 dimension which is trained on PubMed and PMC articles, and word2vec is based on the bacterial abstract training we downloaded from PubMed for 10 years. The experimental results are shown in Fig. 3. As can be seen from the figure, the use of the word embedding in the general domain has a certain effect on the model compared with the random initialization and the performance is better than the model based on machine learning. Also, we can know that the result of using the medical field word vector is better than the general domain word vector, although it is not reach the highest. However, the F1-measure is the best when using the word vector of the bacterial field. As a result, the experiment proves that word vectors in different fields should be used for different professional problems, so that the model effect can be optimal and the error rate will be reduced.

**Fig. 4:**
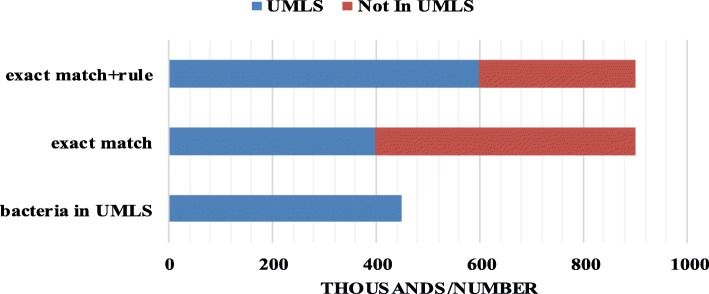
The performance of our model in real dataset

To evaluate the practicability of our model, we utilize the model for named entity recognition on real data. We downloaded more than 400 thousand bacteria-related abstracts from PubMed for bacterial NER, and then compared the identified entity with the bacterial dictionary. UMLS [33] has collected nearly 4.5 million bacterial entities, which is relatively a large database of bacterial entities. Therefore, we extracted all bacterial entities from UMLS to construct a bacterial dictionary. Figure 4 is a comparison of experiments. Compared with 4.5 million bacterial entities in UMLS, more than 500 thousand bacterial entities are not in the dictionary when exact matching; however, when appending some rules, there still have more than 300 thousand entities not in the dictionary. Analyzing the entities predicted by our model shows that even though some predicted entities may be misidentified, our model can still largely predict mainly bacterial strains and bacteria in different ways of writing, and most of them are not updated or included in current dictionary.

## Conclusion and outlook

This paper proposes a method for bacterial named entity recognition based on deep learning and domain features, integrating convolutional neural network, long short-term memory network, and conditional random fields. The experimental results demonstrate that the use of POS features and dictionary features can well promote the recognition of bacterial named entities. At the same time, we also compare the effects of different word embedding on the experimental results. The results illustrate that domain-specific embedding is more effective for bacterial named entity recognition.

Recently, language models have been widely used in the field of natural language, these models have achieved good results in many NLP tasks. In the future, we will combine the language model with bacterial named entity recognition, improve the effect of bacterial named entity recognition, and combine our task with interaction extraction.

## Data Availability

The dataset and model can available at https://github.com/lixusheng1/bacterial_NER.git
